# The threat of artemisinin resistant malaria in Southeast Asia

**DOI:** 10.1016/j.tmaid.2016.11.016

**Published:** 2016

**Authors:** Borimas Hanboonkunupakarn, Nicholas J. White

**Affiliations:** Department of Clinical Tropical Medicine, Faculty of Tropical Medicine, Mahidol University, Bangkok, Thailand; Mahidol-Oxford Tropical Medicine Research Unit, Faculty of Tropical Medicine, Mahidol University, Bangkok, Thailand; Mahidol-Oxford Tropical Medicine Research Unit, Faculty of Tropical Medicine, Mahidol University, Bangkok, Thailand; Centre for Tropical Medicine and Global Health, Nuffield Department of Medicine, University of Oxford, Oxford, United Kingdom

**Keywords:** Malaria, Artemisinin resistance

Fifty years ago it was becoming clear that the enormous global effort to eradicate malaria had failed. There were also an increasing number of worrying reports that the wonder drug chloroquine was not working as it should against falciparum malaria in parts of South-East Asia and South America. Chloroquine resistance spread slowly at first, but by 1979 it had reached the Eastern coastline of Africa, and by 1992 it had crossed the entire continent. Chloroquine could no longer be relied upon to treat malaria, and its preventive efficacy was also in decline. Chloroquine was replaced eventually by sulfadoxine-pyrimethamine as first-line treatment, but this fell rapidly to resistance in many places. Later it was shown by analysis of the sequences flanking the mutant resistance genes (*Pfcrt* and *Pfdhfr* respectively) that the parasites causing illness and death in Africa had their genetic origins close to the Thailand-Cambodia border [1], [2]. In 1984 mefloquine was introduced as first-line treatment for falciparum malaria in Thailand, but resistance soon followed. The prospect of truly untreatable malaria loomed. The region was saved by qinghaosu (artemisinin), a Chinese traditional remedy that has since become the cornerstone of recommended antimalarial treatments [3]. In the treatment of severe malaria parenteral artesunate was shown to reduce mortality substantially and so has become the treatment of choice. Artemisinin-combination therapies (ACTs) are now the first-line treatment for uncomplicated *P. falciparum* malaria throughout the tropical world, and they are increasingly recommended for vivax malaria [3]. But the history of antimalarial resistance emergence and spread is beginning to repeat itself.

Only one year after WHO recommended that ACTs be used everywhere, delayed parasite clearance in *P. falciparum,* suggesting artemisinin resistance, was reported close to the Thailand-Cambodia border [4], [5]. In the following ten years the area in which artemisinin resistance is prevalent has expanded substantially. Artemisinin resistance now extends across the Greater Mekong subregion from the coast of Vietnam in the East to the border of India in the West [6], [7]. There are worrying reports also from French Guiana [8]. To date there is no clear proof that artemisinin resistance has reached Africa yet [9], and no report of artemisinin resistance in other *Plasmodium species* yet. Lack of artemisinin efficacy leaves the ACT partner drug unprotected. Inevitably partner drug resistance (mefloquine, piperaquine) has now followed and recent therapeutic efficacy studies in Thailand and Cambodia show an alarming rise in ACT treatment failure rates [10], [11], [12], [13]. This should be set against a steady reduction in the incidence of malaria in the region. Thus while the risk of treatment failure has risen, the risk of acquiring malaria has fallen.

Artemisinin resistance can be assessed by various methods including measurement of the parasite clearance half-life, a cruder surrogate – the proportion of cases still parasite positive by microscopy at day 3, in-vitro tests assessing ring stage susceptibility, and sequencing of *Pfkelch13* (K13 mutations in the “propeller region” of the gene are associated strongly with resistance) [3], [14]. ACT treatment efficacy is assessed using standard therapeutic efficacy testing of ACTs with 42-day follow up [3]. The updated global distribution of artemisinin resistance can be followed using the K13 Molecular Surveyor, an interactive map provided by the Worldwide Antimalarial Resistance Network (Fig. 1), that shows the current K13 mutation prevalence (http://www.wwarn.org/molecular-surveyor-k13).

Fig. 1. A map of prevalence of K13 mutations from K13 Molecular Surveyor as accessed on 16 November 2016. K13 mutations have been reported from all malaria endemic areas [9]. Some are not associated with artemisinin resistance, and apart from the Greater Mekong subregion of Southeast Asia, there is no evidence that they are being selected elsewhere [15]. This suggests that other genetic changes (collectively termed the “genetic backbone”) contribute to resistance. The great concern is that this genetic backbone and associated K13 mutations will spread to India and Africa. These resistant parasites have already been shown capable of infecting the main African vector *Anopheles gambiae* [16]. Travel between Asia and Africa is very frequent so the spread potential appears high. Recent studies suggest the emergence of a few presumably fitter parasite lineages which have outcompeted the other artemisinin-resistant parasites and spread over long distances. In doing so they have acquired partner drug resistance. The same evolutionary pattern occurred before when chloroquine and sulfadoxine-pyrimethamine resistance spread from the Greater Mekong subregion to Africa [1], [2]. This has led to calls for regional elimination of *P. falciparum* malaria before it spreads to infect the rest of the tropical world [17].

Southeast Asia is not only the epicentre of antimalarial drug resistance. It is one of the most popular travel destinations. Globally, the number of international travelers to the region continues to grow. International tourist arrivals reached 104.3 million in 2015. Thailand, the region's top destination, welcomed 5 million more international tourists in 2015 than it did in 2014. Myanmar, Laos, the Philippines and Indonesia also enjoyed a quick expansion of traveler arrivals. Tourism in Cambodia is slowly yet steadily growing [18]. Visa exemption among ASEAN members also facilitates population movement and, as an unintended by-product, disease movement.

What does all this mean for the traveler? For the vast majority of the malaria-affected world the recommended malaria preventive measures are unaffected. As ever, personal protection from mosquito bites is paramount. In those few forested places where intrepid travelers can acquire malaria in the Greater Mekong subregion, the prophylactic efficacy of atovaquone-proguanil, doxycycline and primaquine should be unaffected by worsening resistance to the ACTs. However, mefloquine will not be as effective as it was previously along the Thailand-Myanmar border. In the treatment of symptomatic falciparum malaria, therapeutic responses to ACTs are slower and failure rates higher, although recent data for artemether-lumefantrine in Thailand and Cambodia are lacking, and elsewhere including most of Myanmar artemether-lumefantrine works well. *P. vivax* remains generally well behaved despite increasing chloroquine resistance. In the absence of further information severe falciparum malaria from areas of known artemisinin resistance should be treated with both artesunate and quinine in full doses. Events are changing rapidly so recommendations for both chemoprophylaxis and treatment should be reviewed frequently.

## Figures and Tables

**Fig. 1:**
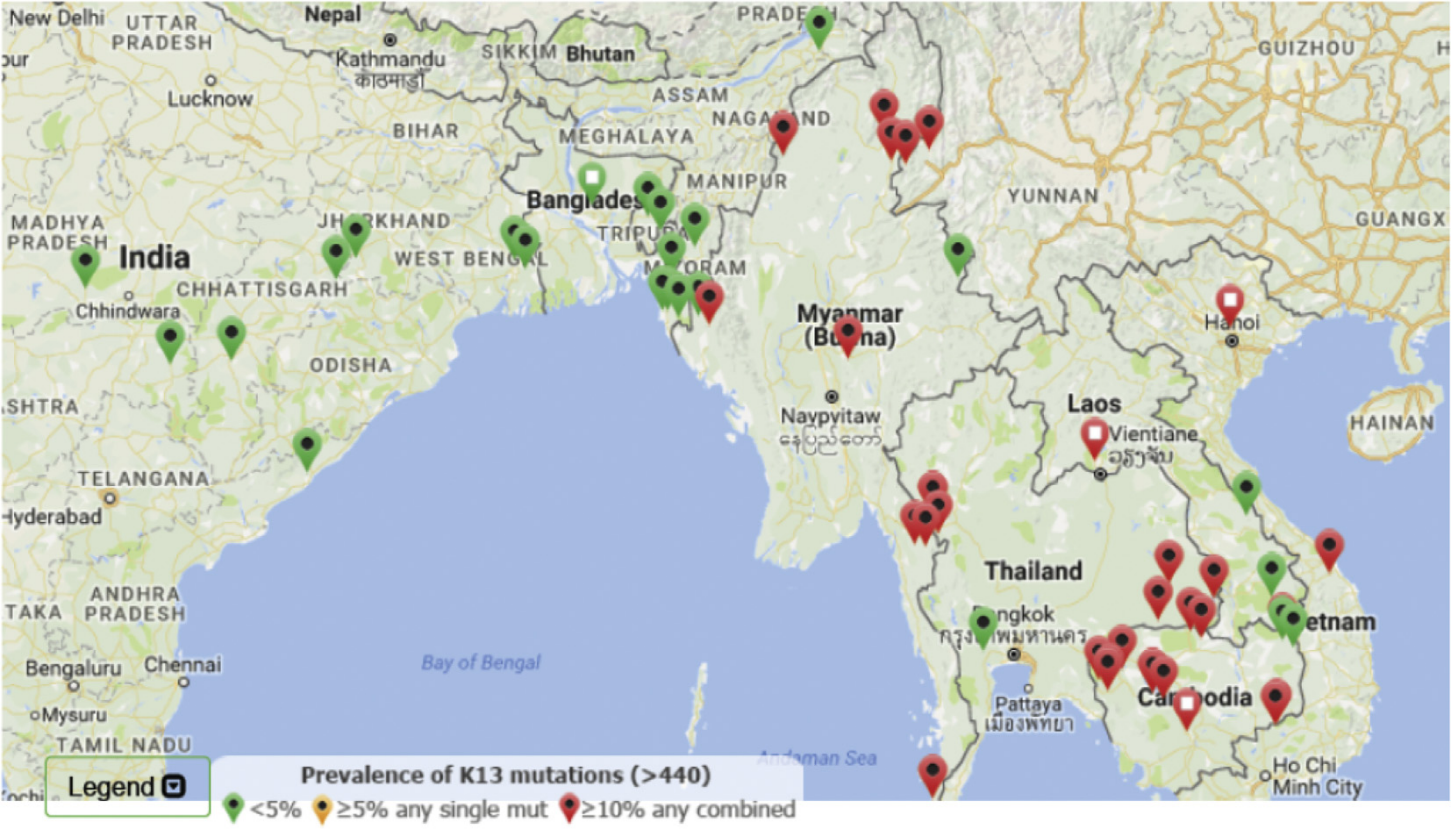
A map of prevalence of K13 mutations from K13 Molecular Surveyor as accessed on 16 November 2016.
